# Complex Gene Loss and Duplication Events Have Facilitated the Evolution of Multiple Loricrin Genes in Diverse Bird Species

**DOI:** 10.1093/gbe/evz054

**Published:** 2019-03-13

**Authors:** Anthony C Davis, Matthew J Greenwold, Roger H Sawyer

**Affiliations:** Department of Biological Sciences, College of Arts and Sciences, University of South Carolina

**Keywords:** epidermal differentiation complex, loricrin, genome, evolution, feathers, scales, birds, reptiles

## Abstract

The evolution of a mechanically resilient epidermis was a key adaptation in the transition of amniotes to a fully terrestrial lifestyle. Skin appendages usually form via a specialized type of programmed cell death known as cornification which is characterized by the formation of an insoluble cornified envelope (CE). Many of the substrates of cornification are encoded by linked genes located at a conserved genetic locus known as the epidermal differentiation complex (EDC). Loricrin is the main protein component of the mammalian CE and is encoded for by a gene located within the EDC. Recently, genes resembling mammalian loricrin, along with several other proteins most likely involved in CE formation, have been identified within the EDC of birds and several reptiles. To better understand the evolution and function of loricrin in birds, we screened the genomes of 50 avian species and 3 crocodilians to characterize their EDC regions. We found that loricrin is present within the EDC of all species investigated, and that three loricrin genes were present in birds. Phylogenetic and molecular evolution analyses found evidence that gene deletions and duplications as well as concerted evolution has shaped the evolution of avian loricrins. Our results suggest a complex evolutionary history of avian loricrins which has accompanied the evolution of bird species with diverse morphologies and lifestyles.

## Introduction

The major event that facilitated the adaptation of amniotes to a fully terrestrial lifestyle was the evolution of a mechanically resilient epidermis which provided a protective barrier and limited water loss to the environment (Chuong 2002). The development of the amniotic epidermis is largely characterized by the cornification of keratinocytes, which represent the main cellular component of the epidermis (Candi et al. 2005). Cornification of keratinocytes is a multistep process that ultimately results in the formation of the terminal layer of the epidermis known as the stratum corneum (SC). The SC confers mechanically resilient properties to the epidermis and its appendages such as hair, nails, and feathers which have aided amniotes in diversifying and inhabiting nearly every ecological niche on the planet, as well as adapt to changing environments, resource availabilities, and climate conditions (Pierard et al. 2000; Strasser et al. 2014).

The SC is composed of terminally differentiated keratinocytes, or corneocytes, in which the plasma membrane has been replaced by an insoluble protein structure known as the cornified envelope (CE). The CE provides mechanically resilient properties such as flexibility and elasticity to the epidermis and its appendages (Candi et al. 2005; Eckhart et al. 2013). The process of CE formation requires strict spatiotemporal regulation of the expression of several different genes and protein substrates (Alibardi et al. 2016). Many of the genes which encode protein substrates involved in CE assembly and structure in mammals are clustered on the human chromosomal region 1q21, which has been termed the epidermal differentiation complex (EDC) (Kypriotou et al. 2012). The EDC of mammals contains genes such as filaggrin, involucrin, and loricrin that are expressed during CE assembly and are critical for proper function of the epidermis and its appendages (Hohl et al. 1991; Robinson et al. 1997; Chuong 2002; Candi et al. 2005). Recently, a genetic locus homologous to the mammalian EDC has been characterized in the chicken and anole lizard, which contains genes of similar exon–intron organization, amino acid composition, and expression profiles (Strasser et al. 2014, 2015). Since then, the EDC locus has been identified in crocodilians, snakes, and turtles that also contain genes characteristic of being involved in the CE assembly. This indicates the EDC locus was present before the divergence of birds and reptiles from mammals. There is no current evidence of an EDC in the genomes of ray-finned fishes (*Takifugu rubripes*), amphibians (*Xenopus tropicalis*, *x. laevis*), or the coelacanth (*Latimeria chalumnae*) supporting the hypothesis that the evolution of the EDC coincided with the adaptation of amniotes to a fully terrestrial lifestyle (Holthaus et al. 2015, 2017).

The main component of the mammalian CE is loricrin, and previous studies have suggested it constitutes 70–85% of the total CE protein content (Hohl et al. 1991; Candi et al. 2005; Eckhart et al. 2013). A more recent study found that while loricrin is a major protein of the CE, they calculated that loricrin has a 11.8–21.5 relative abundance in wild-type mice (Rice et al. 2016). Loricrin is a highly crosslinked structural protein which is extremely rich in glycine as well as polar residues. Studies have found that mutations in loricrin are associated with human skin diseases such as Vohwinkel’s syndrome (VS) and progressive symmetric erythrokeratoderma (PSEK) (Ishida-Yamamoto et al. 1998; Candi et al. 2005). In mammals, loricrin is preferentially crosslinked by transglutaminases (TGases) and provides both elasticity as well as mechanical resistance to the CE (Steinert et al. 1991). Mammals possess a single loricrin gene which contains two exons with the entire coding sequence contained in the second exon. The coding sequence (CDS) is composed of conserved N and C terminal domains rich in lysine and glutamine separated by three central Gly-Ser-Cys-rich repeat domains of variable lengths which are interspersed by short Glutamine-rich regions. This central domain is thought to confer some of the mechanically resilient properties to the CE by taking on a specialized conformation known as the Glycine-loop (Gly-Loop) (Hohl et al. 1991; Steinert et al. 1991). Gly-loops form when at least two quasi-peptide repeats of the form *x*(*y*)_*n*_ are arranged in tandem, where *x* is an aromatic or aliphatic residue, *y* is usually a polar residue (glycine or serine) and *n* is the number of polar residues and is highly variable. The following sequence represents two consecutive Gly-loops in mammalian loricrin, “52-**Y**SGGGG**Y**SGGGGCGGGSSGGGGGGG**I**-76,” which are annotated as *x*(*y*)_5_ and *x*(*y*)_18_ (Hohl et al. 1991). The peptide repeats of mammalian loricrin vary in their exact size and sequence, but all adhere to the *x*(*y*)_*n*_ (Steinert et al. 1991). Sequencing and proteolysis of normal human corneocytes has demonstrated that loricrin is primarily crosslinked to other loricrins via isodipeptide bonds, but loricrin was also found to be crosslinked with small proline-rich proteins (filaggrin and keratin intermediate filaments [KIF]). These crosslinked proteins form a matrix referred to as the KIF-matrix-protein complex. Crosslinking of loricrin with the KIF-matrix-protein complex may provide a means of coordinating cellular structure (Robinson et al. 1997; Wang et al. 2000).

Loricrin has been localized to the EDC in the chicken, two turtles, two snakes, and the anole lizard, however the number of loricrin genes varied across different groups of organisms. Three loricrin genes were identified in the chicken, two in squamates, and only a single loricrin was identified within the EDCs of crocodilians and testudines (Strasser et al. 2014; Holthaus et al. 2015, 2017, 2018). Furthermore, they found that the three chicken loricrin genes are differentially expressed in the beak, scale, comb, claw, feather, and skin of both embryonic and adult individuals (Strasser et al. 2014). Recently, the genomes of several diverse avian species have been sequenced and published allowing researchers to further analyze the conservation and function of avian EDC genes (Strasser et al. 2015; Alibardi et al. 2016). Given the importance of loricrin in the structural properties of the mammalian epidermis as well as loricrins’ expression patterns found in the epidermal appendages of the chicken, studies focusing on loricrins in birds and reptiles may provide insight into the formation of epidermal appendages (Alibardi 2017). To gain a better understanding of the evolution of loricrin genes in birds and reptiles, as well as the roles they play in the development of feathers and scales, we used comparative genomics to screen for loricrin genes in 50 phylogenetically diverse species of birds (Cai et al. 2013; Fankl et al. 2014; Jarvis et al. 2014; Zhang et al. 2014).

## Materials and Methods

### Identification and Characterization of the Epidermal Differentiation Complex in Birds and Reptiles

All genomes were downloaded from the NCBI FTP site in fasta format (supplementary table 1, Supplementary Material online). All genomes had been previously assembled as unplaced genomic scaffolds with the exception of the chicken (*Gallus gallus*) and the zebra finch (*Taeniopygia guttate*) which were assembled at the chromosomal level (Jarvis et al. 2014; Zhang et al. 2014; Yang et al. 2016) BLAST databases of each genome were created using BLAST-2.7.1+ *makeblastdb*. Using the *tblastn* command, each nucleotide database was screened for EDC genes using the amino acid sequences of EDC genes from Strasser et al. (2014) as queries (Altschul et al. 1990, 1997; Pierard et al. 2000; Camacho et al. 2009; Holthaus et al. 2015, 2017). Potential EDC genes identified by *tblastn* searches were extracted using the *blastdbcmd* command as nucleotide sequences in fasta format. These sequences were then translated using the ExPASy translate online analysis tool, and aligned using ClustalW online analysis tools (Thompson et al. 1997; Jeanmougin et al. 1998; Gasteiger et al. 2003).

The genomic organization of avian EDC loci was predicted by aligning identified EDC genes with their respective positions in the chicken. The linearity of DNA sequences was then used to align various genomic scaffolds to recreate each avian EDC region. Several EDC genes, including loricrins, were often not identified by *tblastn* algorithms, however manual screening of genome sequences often found evidence of loricrin genes.

### Phylogenetic Analysis of Loricrins

The loricrin sequences of 15 avian species, 9 mammalian species, 2 crocodilian, 2 testudine, and 3 squamates, which are listed in supplementary Table 2, Supplementary Material online, were used to construct Bayesian and maximum-likelihood (ML) phylogenetic trees. These avian species were selected because they each possess three loricrin genes with both start and stop codons, had no premature stop codons or frameshift mutations, and less than 70% of their central domain was composed of unknown nucleotides (NNNs). Amino acid alignments of loricrin sequences were done using ClustalW2 (Thompson et al. 1997) local alignment tools and edited using Bioedit software (Hall 1999). Using MEGA7 (Kumar et al. 2016), the substitution matrix PROTGAMMAJTTF (JTT+G) was determined to be the best fit substitution model based on Bayesian Information Criterion (BIC), Akaike Information Criterion, corrected (AIC_c_), and the substitution rate (BIC^JTT+G^ = 1299.488, ${\text{AIC}}_{c}^{\text{JTT+}G}$ = 839.458). Bayesian analysis was done using Mrbayes-v3.2 tool (Huelsenbeck and Ronquist 2001; Ronquist and Huelsenbeck 2003). We ran 10,000,000 generations and checked for convergence using the potential-scale reduction factor (PSRF) method (TL: PSRF = 1.0; alpha: PSRF = 1.0) (Gelman and Rubin 1992). ML analysis was performed on the same alignment file using RAxML-v8.2.10 by first using MRE-based bootstrapping until convergence was reached, followed by inferring the best tree produced from generating 1000 thorough ML trees, then mapping the MRE bootstrap values onto the best ML tree (Stamatakis 2014). Generated Bayesian and ML trees were viewed and edited using FigTree-v1.4.3 (Rambaut 2012). Protein sequence alignment (supplementary fig. 2, Supplementary Material online) was generated using T-Coffee online analysis tool (Notredame, Higgins, and Heringa 2000).

### Gene Conversion

Gene conversion analysis was done using GENECONV (Sawyer 1989). Loricrin sequences of only six phylogenetically diverse avian species (*Struthio camelus*, *Manacus vitellinus*, *Chaetura pelagica*, *G.gallus*, *Haliaeetus leucocephalus*, and *Pseuopodoces humilis*) were used due to GENECONV analysis requiring that no NNNs be present in the sequences.

### Prediction and Analysis of Gly-Loop Domains of Avian Loricrins

The Gly-loop domains of six avian species (*G.gallus*, *H.leucocephalus*, *C.pelagica*, *M.vitellinus*, *P.humilis*, and *Melopsittacus undulatus*) as well as the orca (*Orcinus orca*) were predicted using the *x*(*y*)_*n*_ motif described by Hohl et al. (1991). The number and size of Gly-loops of human and mouse loricrins were calculated using the schematic representations proposed by Steinert et al. (1991). Avian species were selected because they were phylogenetically diverse and possessed complete loricrin sequences. Furthermore, with the exception of the budgerigar (*M.undulatus*) (annotated by the asterisks in table 2), they had no NNNs in their central domains. The total number of Gly-loops was predicted by counting the number of gly-ser-rich stretches of sequence present in the central domain ((*y*)_*n*_) that were also bordered by either an aromatic or an aliphatic residue (*x*). Loop sizes were predicted by counting only the number of residues located between aromatic/aliphatic residues which were thought to form gly-loops. The schematic representations of the Gly-loops of chicken LOR3 and LOR1 (fig. 4*A* and *B*) are based on the schematic representations of human and mouse loricrins proposed by Steinert et al. (1991) and are not intended to predict specific secondary structure.

**Table 2:** Table 2 shows the parameters of the respective Loricrin Gly-Loops predicted to form in each gene for 6 bird species (*G. Gallus, H. Leucocephalus, C. vociferus, M vitellinus, P. humilis, and M. undulatus*) and 3 mammals (H. sapien, M. musculus, and O. orca). Mammalian Gly-Loop parameters predicted using LOR sequences deposited in NCBI. Only species with complete central domains were used except for the Budgerigar, whose parameters are indicated by “a”.

	Species	Chicken	Bald Eagle	Killdeer	Manakin	Ground Tit	BUDGERIGAR	Human	Mouse	Orca
Gene	Class	*Galliformes*	*Accipitriformes*	*Charadriiformes*	*Passeriformes*	*Passeriformes*	*Passeriformes*	*Mammalia*	*Mammalia*	*Mammalia*
LOR1	Total no. of loops	27	31	21	23	22	16			
	Largest loop	14	18	20	26	24	19			
	Smallest loop	4	4	4	7	5	4			
	Average loops size	11.44	8.84	12.1	14.78	15.18	9.56			
LOR2	Total no. of loops				11	17	18^a^			
	Largest loop				30	27	26^a^			
	Smallest loop				6	7	3^a^			
	Average loops size				21.64	17.41	13^a^			
LOR2B	Total no. of loops						27^a^			
	Largest loop						25^a^			
	Smallest loop						3^a^			
	Average loops size						7.6^a^			
LOR3	Total no. of loops	43	10	33	34	34				
	Largest loop	26	25	25	21	17				
	Smallest loop	3	3	4	2	2				
	Average loops size	11.44	11.5	10.64	8.29	8.03				
LOR3B	Total no. of loops	48	8	43						
	Largest loop	26	17	27						
	Smallest loop	3	7	4						
	Average loops size	11.4	11.25	11.58						
Mammalian	Total no. of loops							21	22	6
	Largest loop							18	34	37
	Smallest loop							2	11	3
	Average loops size							10.62	18.18	16.67

a Indicates NNNs in the central domain.

### Amino Acid Composition and Statistical Analysis of Loricrin

Amino acid analysis was performed using avian loricrin sequences classified as complete and which were composed of <15% NNNs’s, as well as mammalian, crocodilian, and squamate loricrin sequences. Translated amino acid loricrin sequences were analyzed for amino acid composition using ExPASy ProtParam tool (cite ExPASy). In order to account for the large amount of variation in size observed across loricrin genes, all amino acid analyses were done using the percentage of each amino acid present in the sequence as opposed to the total number of residues. The resulting percentage of each amino acid residue for each loricrin sequence analyzed can be found in supplementary table 4*A* and *B*, Supplementary Material online. These data were used to generate the principal component analysis (PCA) in R (fig. 5) by means of the BiocLite-pcaMethods package by BioConductor. The PCA was done using thing singular-value decomposition (SVD) method.

Further amino acid analyses were performed by comparing the percentage of each of the 20 amino acid residues observed across the respective loricrins of each species examined in order to identify significant differences in the amino acid contents of respective amino acid residues. Significance was determined using analysis of variance (ANOVA) and Welch’s *t*-test analysis which was performed using Microsoft Excel: Data Analysis ToolPak.

## Results

### Loricrin Conservation within the EDC across Birds and Reptiles

In order to establish whether the loricrin genes identified in the chicken and anole lizard by Strasser et al. (2014) are conserved across birds and reptiles, we screened the genomes of 2 crocodilian species (*Alligator mississippiensis* and *Crocodylus porosus*) and 50 phylogenetically diverse avian species (supplementary table 1, Supplementary Material online) using the amino acid sequences of the chicken, king cobra, burmese python, chinese soft-shelled turtle, western painted box turtle, and the anole lizard EDC genes as BLAST queries (Altschul et al. 1990, 1997; Pierard et al. 2000; Camacho et al. 2009; Holthaus et al. 2015, 2017). Bird genomes searched in this analysis came from the recently sequenced genomes of 48 diverse bird species (Jarvis et al. 2014; Zhang et al. 2014). We also searched the genomes of the ground tit (*P.humilis*) and Atlantic canary (*Serinus canaria*) (Cai et al. 2013; Fankl et al. 2014). All genomes were obtained from NCBI and were previously assembled at the scaffold level with the exception of the chicken, zebra finch, and turkey which were assembled to the chromosome level. Identified loricrin (LOR) genes were added to the query file and iterative rounds of BLAST searches were performed on the avian genomes.

**Fig. 1.**
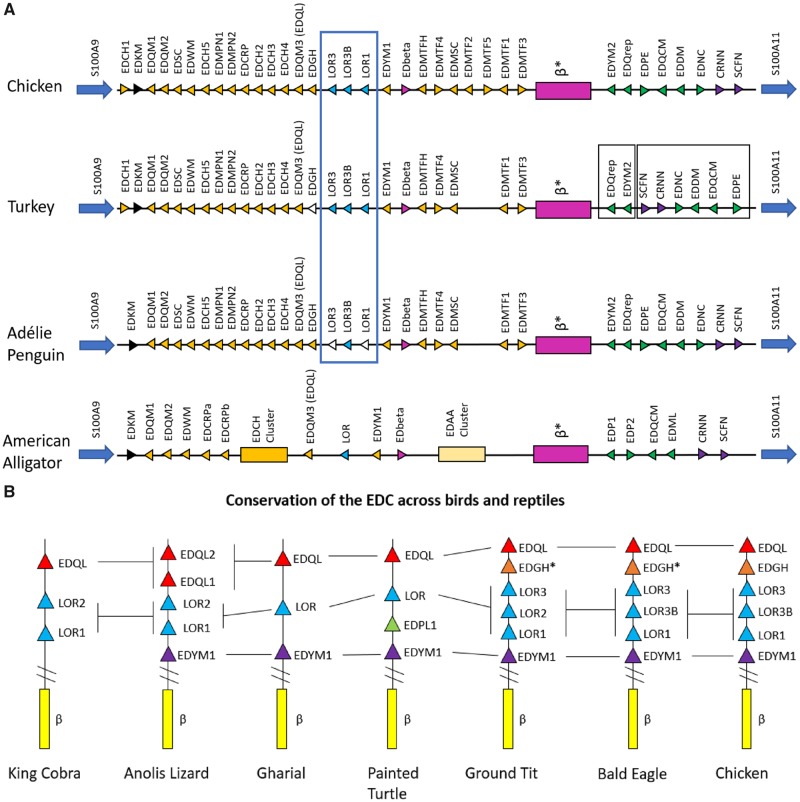
—Genomic organization of loricrin within the EDC of archosaurs. (*A*) Schematic overview of the conservation of the entire EDC of the chicken (*G. gallus*), turkey (*Meleagris gallopavo*), adélie penguin (*Pygoscelis adeliae*), and saltwater crocodile (*C. porosus*). Chicken EDC organization identical to that proposed by Strasser et al. (2014), with the exception of the identification of EDMTF5. Filled-in arrows with black outlines represent complete SEDC genes, arrows with white fills indicate incomplete genes. Gene number annotations LOR1, LOR2, LOR3 come from annotations of chicken loricrins by Strasser et al. (2014). Colors correspond to classifications by Strasser et al. (2014). (*B*) Schematic representation of the region of the EDC which contains loricrins between the conserved genes EDQL (formerly EDQM3) and the β-keratin gene cluster. The genes EDQL and EDYM1 are conserved across all species examined. EDGH sequences were identified in all avian species, however, the start codon from the chicken identified by Strasser et al. (2014) was not present in other bird species(*). The loricrin copy number varied across different groups of organisms, but in general squamates possessed 2, crocodilian and testudine species contained 1, and birds 3 loricrin genes. Arrow colors represent related genes. Parallel lines between EDYM1 and β-box indicate presence of variable number of lineage-specific EDC genes. King cobra EDC genes identified by Holthaus et al. (2016), anole lizard EDC genes identified by Strasser et al. (2014), and painted turtle EDC genes identified by Holthaus et al. (2015).

The results of these BLAST searches confirmed evidence of at least a single copy of loricrin in the two crocodilian species and the 50 bird species (supplementary table 1, Supplementary Material online). When multiple loricrin genes were identified in the bird genomes, we found them to be tandemly arranged in the same orientation and conserved within the EDC between the EDGH and EDYM1 genes (fig. 1). We found evidence of only a single loricrin gene in the crocodilian genomes, which is in agreeance with a recent study characterizing the crocodilian EDC (Holthaus et al. 2018). Previous studies found a single loricrin gene in turtles whereas two loricrin genes are present in squamates (fig. 1) (Holthaus et al. 2015, 2017; Strasser et al. 2014). In birds, evidence of three loricrin genes was identified in 39 of the 50 species examined, however, in many species this region of the EDC (in which loricrins are located) was either incomplete (assembled across multiple scaffolds) or composed almost entirely of unknown nucleotides (NNN’s) (supplementary fig. 1*A* and *B*, Supplementary Material online). This resulted in only the ground tit (*P.humilis*), bald eagle (*H.leucocephalus*), and chicken (*G.gallus*) having three uninterrupted, complete loricrin sequences (table 1).

**Table 1:** Quality of Avian Loricrin Gene Sequences Identified in Current Draft Genomes

Bird	LOR3	**LOR2** * **LOR3B**	LOR1
Zebra finch	3	9^a^	1
Medium ground finch	8	3^a^	2
Ground tit	1	1^a^	1
Atlantic canary	1	2^a^	3
American crow	5	5^a^	7
Hooded crow	1	6^a^	1
Golden-collared manakin	1	1^a^	7
Budgerigar	2	3^a^	2
Peregrine falcon	3	1	1
Bald eagle	1	1	1
Little egret	6	1	2
Crested ibis	3	3	1
Adélie penguin	3	1	2
Emperor penguin	4	3	7
Killdeer	2	2	1
Hoatzin	3	3	3
Anna’s hummingbird	1	2	1
Chimney swift	1	8	1
Common cuckoo	5	4	3
Downy woodpecker	4	3	1
Pigeon	1	Not found	1
Chicken	1	1	1
Duck	5	5	2
White-throated tinamou	3	4	1
Ostrich	1	1	2

Note.—(1) 100% complete gene, (2) contained no more than 15% unknown nucleotides (NNNs) in the central domain, (3) contained between 15.1% and 75% NNNs in the central domain, (4) contained between 75% and 90% NNNs in the central domain, (5) indicates either all or part of the C-terminal region was absent (including stop codon); (6) indicates either all or part of the N-terminal region was absent (including start codon), (7) indicates the presence of frameshift mutation(s), (8) contained a premature stop codon; (9) indicates that evidence of a loricrin gene was identified however it represented less than 10% of the complete sequence of its orthologue in the chicken (*G. gallus*).

^a^Indicates the gene is LOR2 versus LOR3B.

In order to analyze the number of loricrin genes conserved across birds, we narrowed our results by selecting species in which the loricrin containing region of the EDC (fig. 1*B*) was assembled on a single scaffold. Twenty-five phylogenetically diverse avian species (table 1) were found to have this portion of the EDC; however, 22 of these species still possessed loricrin sequences containing NNN’s. We found evidence suggesting the presence of three loricrin genes in all but one (pigeon) of these 25 species (table 1). The pigeon (*Columbia livia*) was found to have only two loricrins with no evidence of a third loricrin. We did not find evidence suggesting the presence of more than three or less than two loricrin genes in any of these 25 bird species.

### Phylogenetic Analyses Suggest a Complex and Dynamic Evolutionary History of Loricrins in Birds

**Fig. 2.**
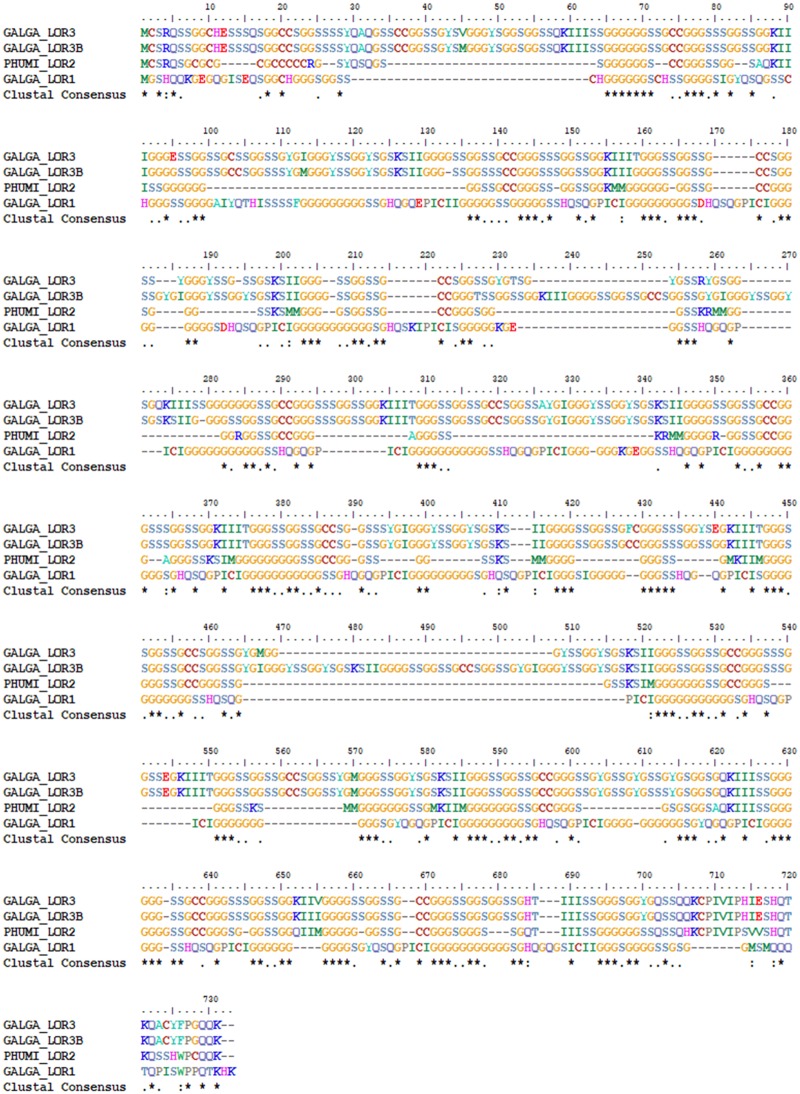
—Sequences of identified loricrin genes of chicken (GALGA; LOR1, LOR3, LOR3B) and ground tit (PUMI; LOR2). The identified sequences of loricrins identified in chicken (GALGA; LOR1, LOR3, LOR3B) and ground tit (PHUMI; LOR2). LOR1 contains unique N- and C-terminal sequences and a unique repeat unit compared with other loricrins. LOR3 and LOR3B are identical in sequences and differ only in individual amino acid substitutions and number of repeat units. LOR2 is found only in passerine birds and is highly similar to LOR3/LOR3B with the exception of the identification of aromatic/aliphatic residues in the repeat and a small cysteine-rich stretch of amino acids at the N-terminus.

**Fig. 3.**
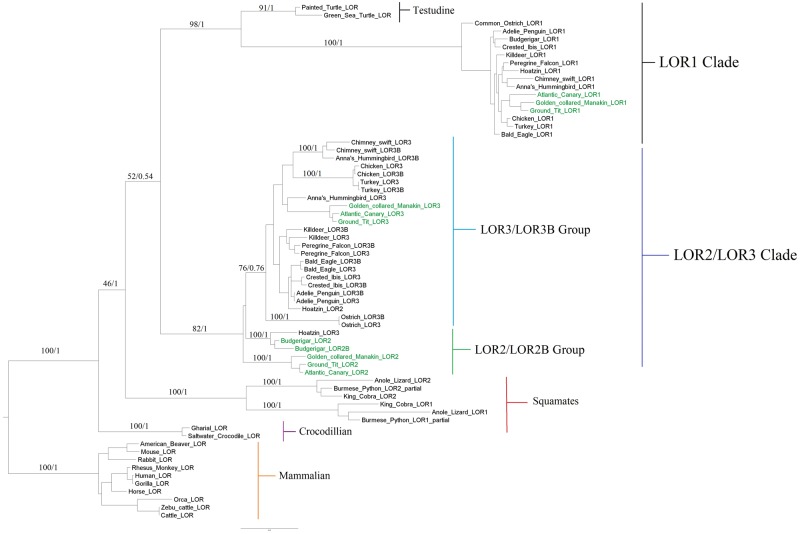
—Maximum liklihood (ML) analysis of loricrin sequences. Phylogenetic tree generated by ML analyses. This tree is largely in agreeance with our baysian analysis tree (supplementary fig. S3, Supplementary Material online). Nonavian loricrins formed three distinct clades consisting of mammals, squamates, and crocodilian loricrin sequences respectfully. In contrast to currently accepted comprehensive phylogenetic data, our phylogeny places crocodilians as the basal group to all birds and reptiles. Avian loricrins were organized into two major clades. The first LOR1 clade included all terminal avian loricrins that bordered EDYM1 annotated as LOR1 as well as testudine loricrins as a sister group. The second avian clade was LOR2/LOR3 clade which consisted of two major sister groups of LOR3 and LOR2 respectfully. Only passerine birds and the Hoatzin possessed LOR2 loricrins. All species possessed a LOR3 loricrin, and all species with the exception of Passeriformes, the hoatzin and Anna’s Hummingbird possessed a LOR3B gene organized in a lineage specific manner.

Similar to the mammalian loricrin, avian loricrins are composed of highly conserved N- and C- terminal domains separated by a highly variable glycine-rich repeat domain (fig. 2 and supplementary fig. 2, Supplementary Material online) (Hohl et al. 1991). Likely due to the highly repetitive nature of loricrins, many loricrin genes did not assemble well in the avian genomes and are composed of unknown nucleotides (NNNs) (Milinkovitch et al. 2010; Hron et al. 2015). Therefore, we used specific parameters to screen loricrin genes for inclusion in phylogenetic analyses. Loricrin sequences were considered complete provided that: (1) the N- and C- termini were both present without any NNNs, (2) within the central domain, at least three tandemly arranged repeat units are present without NNNs, and (3) no more than 15% of the central domain contained NNNs. Loricrin sequences in compliance with (1) and (2), but contained >15% but less than 70% NNNs were considered partial sequences. This resulted in 15 avian species having 3 complete or partial loricrin genes which were used in phylogenetic analyses (supplementary table 2 and fig. 3, Supplementary Material online, fig. 3). In addition to these 15 avian species and their three loricrin genes, we included a single loricrin gene from nine mammals, two loricrin copies from two snakes and a lizard (Holthaus et al. 2017), one loricrin from two turtle species (Holthaus et al. 2015) and a single loricrin we identified from the two crocodilian species (supplementary table 2, Supplementary Material online) in the phylogenetic analyses.

Bayesian (supplementary fig. 3, Supplementary Material online) and ML (fig. 3) analyses were performed using MrBayes v. 3.2 (Huelsenbeck and Ronquist 2001; Ronquist and Huelsenbeck 2003) and RAxML v. 8.0.0 (Stamatakis 2014). The topology of these phylogenies largely agreed with a few exceptions related to node support values. The nine loricrins of mammals were used to root the phylogeny with the reptile and avian loricrins forming a well-supported monophyletic clade. Due to a low bootstrap value in the ML phylogeny (fig. 3), the reptile and avian loricrins are composed of four monophyletic clades comprising a crocodilian clade, squamate clade, testudines and avian loricrin 1 clade and an avian loricrin 2 and 3 clade. In contrast, a high posterior probability support value indicates that the crocodilian clade is the outgroup to all other reptile and avian loricrins (fig. 3). These results conflict with the currently accepted topology of reptiles and birds which indicates that crocodilians and birds form the monophyletic clade of archosaurs and that squamates (excluding tuatara) are the outgroup to other reptiles (turtles, crocodilians) and birds (Crawford et al. 2012; Miller et al. 2012).

**Fig. 4.**
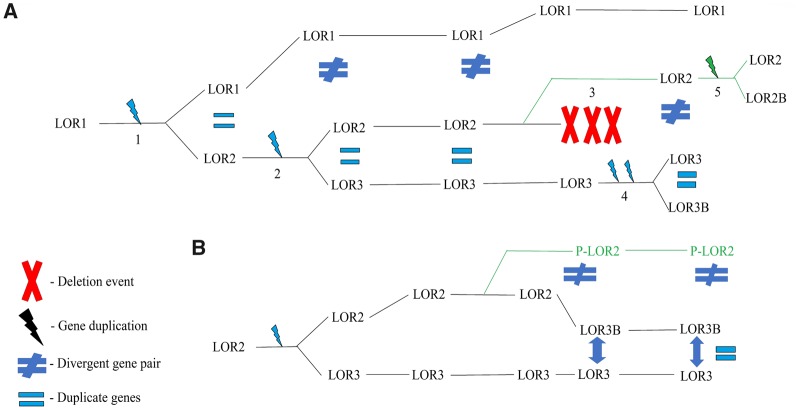
—Schematic of possible scenario detailing evolutionary history of avian loricrins. (*A*) (1) Duplication of the ancestral loricrin gene (LOR1) resulted in two copies of loricrin (LOR1 and LOR2) before the emergence of the crown birds (Prum et al. 2015). (2) Duplication of LOR2 resulted in LOR2 and LOR3 genes. (3) Following the divergence of Passeriformes, deletion of LOR2 in other major orders of birds resulted in a single copy of loricrin (LOR3) in most orders of birds whereas LOR2 was retained in Passeriformes. (4) In nonpasserine lineages, LOR3 duplicated and produced lineage-specific LOR3B found in Palaeognathae, Galloanserae, and Neoaves (excluding Passeriformes) species. (5) In the case of Psittacisformes, a sister clade of Passeriformes, the retained LOR2 duplicated and produced Psittaciforme-specific LOR2B in budgerigar. (*B*) Depicts a second scenario where the similarities between LOR3 and LOR3B are the result of concerted evolution of an ancestral duplication as opposed to similarity resulting from a recent duplication. Identical to scenario 1 until following duplication of LOR2. Ancestral LOR2 and LOR3 duplicate genes undergo gene conversion events resulting in concerted evolution. Evolution of LOR2 in Passeriformes (P-LOR2) resulted in its divergence. Continued concerted evolution in nonpasserine birds has maintained nearly identical loricrin paralogs.

The squamate loricrin clade consists of two subclades composed of a squamate loricrin 1 gene and a squamate loricrin 2 gene indicating that a duplication occurred early in squamate evolution. Interestingly, the avian loricrin 1 and testudine loricrin genes form a monophyletic clade possibly indicating convergent evolution. In contrast, this may indicate that the avian loricrin 1 gene is highly conserved and represents the ancestral loricrin of turtles and archosaurs. The final clade (LOR2/LOR3 Clade; fig. 4) of avian loricrins consists of multiple loricrin copies with a dynamic duplication history.

The LOR2/LOR3 clade (fig. 3) containing avian loricrins was made up of two major sister groups. One of these sister groups (LOR2; fig. 3), contained passerine loricrin sequences as well as a single loricrin gene belonging to the Hoatzin (OPHHO; *Ophisthocomus hoazin*). While most of the passerine species had only one loricrin gene in this clade (LOR2), the budgerigar (MELUN; *M.undulatus*) had two copies which were annotated as LOR2 and LOR2B. The other sister group (LOR3/LOR3B) contained representatives from all species including passerines, however the latter only contained a single loricrin gene while the former all contained two copies which displayed a lineage-specific duplication history. These Loricrin sequences were designated as LOR3 and LOR3B (fig. 3). LOR2B and LOR3B, or “B-type” loricrins, were nearly identical to their paralogous LOR2 and LOR3 gene, respectively.

The loricrin genes of the Hoatzin (OPHHO; *O.hoazin*) and Anna’s Hummingbird (CALAN; *Calypte anna*) displayed unique evolutionary histories relative to other avian species’ LOR2 and LOR3 sequences. The hoatzin was the only nonpasserine bird which possessed a loricrin gene in the passerine LOR2 sister group. The hoatzin’s other loricrin gene was closely related to the LOR3/LOR3B gene of the adélie penguin, bald eagle, crested ibis, peregrine falcon, and killdeer. In the case of Anna’s Hummingbird, one loricrin gene formed a sister group with both chimney swift loricrin genes (LOR 3 and 3B) and the other formed a sister group with LOR3 of passerine birds. Our phylogenetic results within the LOR1 clade and the LOR2 and LOR3 clade were largely in agreeance with recent comprehensive avian phylogenies proposed by Prum et al. (2015) which places the enigmatic Hoatzin as a sister group to other landbirds (Ericson et al. 2002; Jarvis et al. 2014; Prum et al. 2015) (fig. 3).

The results of these phylogenetic analyses suggest two possible scenarios for the evolution of avian loricrins. The first scenario is detailed in figure 3 and involves multiple lineage-specific duplications and deletions where (1) duplication of the ancestral loricrin gene (Anc_LOR) resulted in two copies of loricrin (LOR1 and LOR2) before the emergence of the crown birds (Prum et al. 2015). (2) Duplication of LOR2 resulted in LOR2 and LOR3 genes. (3) Following the divergence of Passeriformes, deletion of LOR2 in all other major orders of birds resulted in a single copy of loricrin (LOR3) in most orders of birds whereas LOR2 was retained in Passeriformes. (4) In nonpasserine lineages, LOR3 duplicated and produced LOR3B found in Palaeognathae, Galloanserae and Neoaves (excluding Passeriformes) species. (5) In the case of Psittacisformes, a suborder of passerine birds, the retained LOR2 duplicated and produced Psittaciforme-specific LOR2B in budgerigar. No evidence was found of a fourth loricrin gene in Psittaciformes suggesting that the LOR3 present in other birds may have been lost in this lineage (figs. 3 and 4).

**Fig. 5.**
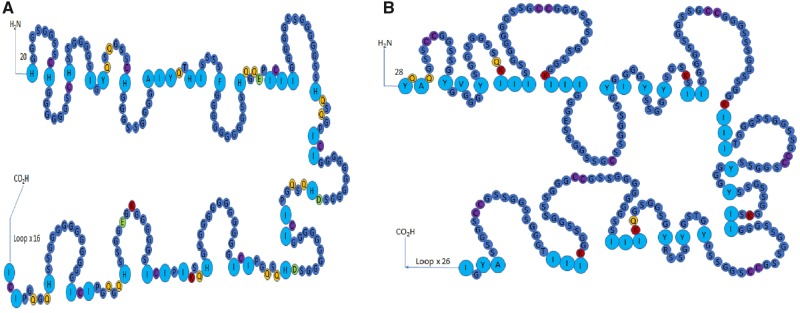
—Schematic representation of the central domain of LOR Gly-loops in the chicken. (*A*) Chicken LOR1 likely contains extended arrays of glycine loops which, similarly to mammalian loricrins, are interspersed by glutamine rich domains of different structures. This representation does not infer any particular three-dimensional arrangement of the loops, but since mammalian loricrins are known to contain N-(γ-glutamyl)—lysine isodipeptide bonds, it is likely loricrins adopt a compact rosette-like structure. (*B*) There are 43 total predicted loops in GALGA LOR3. The loops range in size from 3 to 26 residues indexed on aromatic/aliphatic residues. Conserved adjacent cysteine residues are located at the apex of several of the larger loops and possible participate in disulfide bonding. This schematic does not infer any further three-dimensional structure of the loops. Glutamine and lysine residues are also located at conserved positions throughout the sequence. Loops are generally indexed upon dimers/trimers of aliphatic residues or lone aromatic residues.

The second possible scenario is that concerted evolution of LOR2B and LOR3B with LOR2 and LOR3, respectively, has resulted in the phylogenetic distribution of loricrin paralogs (fig. 4). This second scenario may have occurred though gene conversion, a mechanism of concerted evolution. Gene conversion events occur through unequal recombination where a stretch of DNA is replaced by a homologous region such as those found in duplicate genes that results in the homogenization of both genes (Daiquing 1999). We used GENECONV (Sawyer 1989) to assess the likelihood that gene conversion led to concerted evolution of LOR3/LOR3B. Due to complications associated with incomplete sequences and NNN’s, we were left with seven diverse avian species (chicken, ostrich, ground tit, chimney swift, golden-collared manakin, bald eagle, Atlantic canary) which contained complete loricrin genes and no NNN’s. The results of the GENECONV analysis found strong evidence of a gene conversion event between LOR3 and LOR3B for one species, the chimney swift (*C.pelagica*) (BC KA, *P* = 0.00213), which possessed a 91 nucleotide long global fragment that contained 43 polymorphic sites. No other significant gene conversion events were detected between LOR2/LOR2B and LOR3/LOR3B in the other species (supplementary table 3, Supplementary Material online). These results support scenario one, which is detailed in figure 5.

### Avian Loricrin Genes Form Gly-Loops of Variable Size and Number

The central domain of mammalian loricrin is thought to take on a specialized structural conformation termed the Gly-loop which results from tandemly arranged quasi-repetitive, glycine-rich peptide sequences. The Gly-loop conformation is a key structural motif which contributes to the elasticity of the epidermis as well as its ability to form an insoluble barrier to the external environment, which was a crucial evolutionary event (Hohl et al. 1991; Candi et al. 2005). The properties conferred by the Gly-loop motif depend heavily on the relative composition of amino acids which make up the peptide repeats of the central domain as well as the presence of specific residues in the N- and C- termini (Steinert et al. 1991). Here, we calculated the size and total number of Gly-loops for six diverse avian species as well as three mammals (table 2). This allowed us to analyze the interspecific and intraspecific amino acid variation of avian Gly-loops as well as the variation in the number and size of avian Gly-loops.

We found that the repetitive units which comprise the central domain of avian loricrins conform to the general form (*x*(*y*)_*n*_) required for the formation of Gly-loops and that there is a significant amount of variation in the amino acid composition and organization of avian loricrins. In general, there are distinct amino acid differences between Gly-loops formed by the avian loricrin genes. The *x*(*y*)_*n*_ sequences of the two Gly-loops predicted to be formed in the loricrins of the chicken are “133-**II**GGGGGSSGGGGGSSHQSQGP**I**C**I-**158” for LOR1 and “128-**II**GGGGSSGGSSGCCGGGSSSGGSSGGK**III**-158” for LOR3/3B (fig. 2). The sequence of a predicted Gly-loop in LOR2 of the ground tit (*P.humilis*) was “156-**MM**GGGGGGGGSSGCCGGGSGGGSSKS**MM-**202” (fig. 2). LOR1 Gly-loops are interspersed by glutamine and proline residues and are indexed primarily on aliphatic isoluecines, or the “*x*” of the *x*(*y*)_*n*_ conformation (fig. 5*A*). The Gly-loops of LOR2 and LOR3 are interspersed by conserved lysine and cysteine residues. However, LOR2 loops are indexed primarily on aliphatic methionines, while LOR3 loops are indexed on either tyrosine or isoluecine residues (fig. 4*B*). B-type loricrins (LOR2B and LOR3B) conform to the same general Gly-loop amino acid characteristics as their duplicates LOR2 and LOR3.

Although all avian loricrins conform to the general form *x*(*y*)_*n*_ (fig. 2), we observed considerable variation in the number and the size of Gly-loops (table 2). As previous studies (Hohl et al. 1991; Steinert et al. 1991) have shown, we found that mammalian loricrins vary extensively in both size and number of Gly-loops. The variation observed across mammalian loricrins is thought to result in slight differences in the mechanical properties exhibited by the CE (Steinert et al. 1991).

Similar to mammalian loricrins, we observed significant variation in the size and number of Gly-loop domains of avian loricrins. Out of six avian species analyzed, the longest Gly-Loop contained 30 residues between “*x*” residues (*x*(*y*)_*n*_) (MANVI LOR2) and the shortest contained two (MANVI + PHUMI LOR2). The highest amount of interspecific variation in the number of Gly-loops was in LOR3/LOR3B, where the total number of predicted Gly-loops ranged from 8 in LOR3B of the bald eagle to 48 in LOR3B of the chicken (table 2). While there is considerable interspecific variation in the total number of Gly-loops making up LOR3/LOR3B, the size parameters of those loops were more conserved (LOR3: average loop size = 10.52, SD = 1.48, *n* = 8) relative to the size parameters of the loops of other avian loricrins (LOR1: average loop size = 11.98, SD = 2.61, *n* = 6; LOR2: average loop size = 19.52, SD = 2.99, *n* = 2) (table 2). Despite the high amount of interspecific variation observed across the size and number of Gly-loops in LOR3/LOR3B, there was relatively little variation observed within species. For instance, LOR3 and LOR3B of the chicken are predicted to contain 43 and 48 total loops respectively, whereas LOR3 and LOR3B of the bald eagle are predicted to contain 10 and 8 loops, respectively (table 2). Overall, these results demonstrate that avian loricrins, much like mammalian loricrins, exhibit a large amount of variation in the size and number of the Gly-loops even between closely related species, but also that birds which exhibit different lifestyles, such as predatory and domestic, have differences in the properties and numbers of their Gly-loops. Due to uncertainty with the number of actual NNNs present in incomplete avian loricrin genes, our analysis was restricted to a small sample size (*n* = 6). Therefore, more complete avian loricrin sequences are needed to make inferences relating the size and number of Gly-loops to functional properties of avian loricrins.

### Amino Acid Compositional Differences between Avian Loricrin Genes Suggests Functional Diversity

Similar to mammalian loricrins, the amino acid composition of avian loricrins are extremely biased with over 50% of the gene being composed of glycine and serine (supplementary table 4*A* and *B*, Supplementary Material online). Other prevalent amino acids are cysteine, tyrosine, lysine, and glutamine, which are all associated with protein cross-linking (Hohl et al. 1991; Steinert et al. 1991; Candi et al. 2005; Eckhart et al. 2013).

**Fig. 6.**
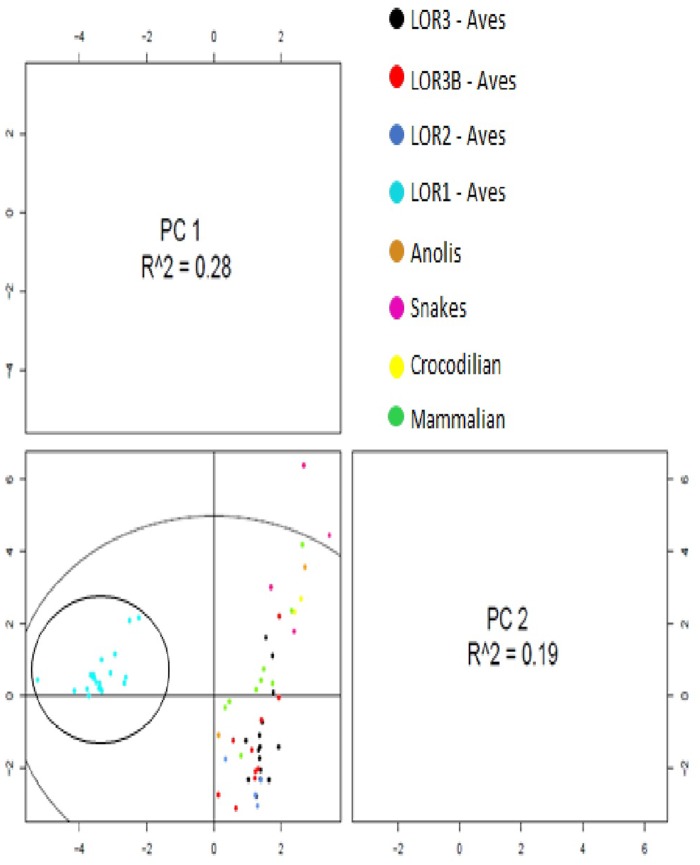
—PCA of loricrin sequences. PCA generated in R, BiocLite-pcaMethods package using the SVD method. Respective loricrin sequences are indicated by color as represented in the key. The black circle surrounds the avian LOR1 cluster. The amino acid contents of avian LOR1 were unique relative to all other loricrin sequences. All other loricrin genes including LOR2, LOR3, and LO3B of Aves failed to sort into distinct groups, highlighting the large amount of diversity observed across loricrin sequences. PCA plot was able to explain 46.79% (PC1 = 0.2764%, PC2 = 0.1915%) of the total variance between the amino acid contents of loricrin sequences.

**Fig. 7.**
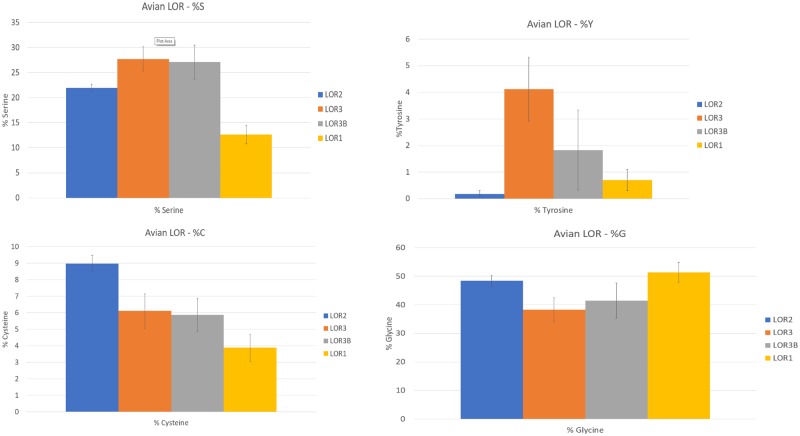
—Significant variation in amino acid residues associated with epidermal development and structure across avian loricrins. In clockwise order starting at the top left: the average percentage of serine (S), tyrosine (Y), Cysteine (C), and Glycine (G) across avian loricrins. For all four residues pictured, there were significant differences (**P < 0.001) observed in LOR2 and LOR1 from other loricrins (Significant differences for all blue and yellow bars.). Data from AA analysis in supplementary table 4*A* and *B*, Supplementary Material online. Respective LOR orthologues distinguished by color and from left to right: LOR2, LOR3, LOR3B, LOR1.

In order to further assess the potential functional properties of avian loricrins, we analyzed the amino acid composition of all loricrin sequences identified as having less than 10% NNNs. Using the ExPASy ProtParam tool (Gasteiger et al. 2003), we calculated the percent composition of the 20 amino acids for 48 avian, 8 reptilian, and 9 mammalian loricrin genes (supplementary table 4*A* and *B*, Supplementary Material online). Using these data, we generated a PCA using the Bioconductor pcaMethods package in R (Stacklies et al. 2007; RStudio Team 2015). The PCA plot (fig. 6) was able to explain 46.79% (PC1 = 0.2764%, PC2 = 0.1915%) of the total variance between the amino acid composition of loricrin sequences. The PCA also found that principle component 1 (PC1) differentiated avian LOR1 into a distinct cluster relative to all other loricrin genes. The amino acid composition of the remaining loricrin sequences failed to sort into unique clusters; however, LOR2 and LOR3 of birds did group together but could not be differentiated from one another. The loricrins of crocodilians, snakes, and some mammals increased the vertical spread (PC2). Overall, these results demonstrate that avian LOR1 has a conserved and unique amino acid composition, while avian LOR2 and LOR3/LOR3B loricrins could not be differentiated from reptilian and mammalian loricrin genes (figs. 6 and 7). Together with our phylogenetic results (figs. 3 and 4), these results suggest that avian LOR1 diverged early in the evolution of birds and has remained conserved within birds.

To characterize which amino acid residues were primarily contributing to the PCA analysis results, we performed an ANOVA to analyze the differences of the mean amino acid content between avian loricrin genes. We observed statistically significant differences in seven amino acid residues between LOR1 and LOR2 and 11 amino acid residues between LOR1 and LOR3 (supplementary table 5, Supplementary Material online). The most significant amino acid differences between LOR1 from LOR2 and LOR3 were observed in serine (LOR1: $\bar{X}$ = 12.64%, *n* = 17; LOR2: $\bar{X}$ = 21.86%, *n* = 4, *F*_17, 5_ = 119.59, *P* < 0.001; LOR3: $\bar{X}$ = 27.79%, *n* = 15, *F*_17, 14_ = 372.9, *P* < 0.001), cysteine (LOR1: $\bar{X}$ = 3.88%, *n* = 17; LOR2: $\bar{X}$ = 8.82%, *n* = 4, *F*_17, 5_ = 156.83, *P* < 0.001; LOR3: $\bar{X}$ = 6.12%, *n* = 15, *F*_17, 14_ = 43.15, *P* < 0.001) and proline (LOR1: $\bar{X}$ = 3.38%, *n* = 17; LOR2: $\bar{X}$ = 0.66%, *n* = 4, *F*_17, 5_ = 222.51, *P* < 0.001; LOR3: $\bar{X}$ = 1.07%, *n* = 15, *F*_17, 14_ = 166.63, *P* < 0.001).

Our phylogenetic analyses (fig. 3) found that with the exception of the Hoatzin, only passerine birds possess LOR2. However, most avian species, except Budgerigar, possess a LOR3 gene. In order to determine if the passerine LOR2 (P-LOR2) and passerine LOR3 (P-LOR3) differ in amino acid composition (unlike LOR3/LOR3B of nonpasserine birds); we performed an ANOVA analysis between LOR2 and LOR3 using either only passerine genes or only nonpasserine genes (NP). Only tyrosine content (P-LOR3: $\bar{X}$ = 5.68%, *n* = 6; LOR3-NP: $\bar{X}$ = 3.08, *n* = 9; *F*_6, 9_ = 10.14, *P* < 0.01) was found to significantly differ between P-LOR3 and LOR3 of nonpasserine birds indicating they have nearly identical amino acid compositions. In contrast, significant differences were observed in cysteine (P-LOR2: $\bar{X}$ = 8.82%, *n* = 5; P-LOR3: $\bar{X}$ = 5.87%, *n* = 6; *F*_5, 6_ = 27.3, *P* < 0.001), glycine (P-LOR2: $\bar{X}$ = 47.76%, *n* = 5; P-LOR3: $\bar{X}$ = 40.57%, *n* = 6; *F*_5, 6_ = 12.76, *P* < 0.01), serine (P-LOR2: $\bar{X}$ = 21.86%, *n* = 5; P-LOR3: $\bar{X}$ = 26.57%, *n* = 6; *F*_5, 6_ = 18.42, *P* < 0.001), tyrosine (P-LOR2: $\bar{X}$ = 0.22%, *n* = 5; P-LOR3: $\bar{X}$ = 2.08%, *n* = 6; *F*_5, 6_ = 50.24, *P* < 0.001) and valine (P-LOR2: $\bar{X}$ = 0.54%, *n* = 5; P-LOR3: $\bar{X}$ = 2.08%, *n* = 6; *F*_5, 6_ = 25.2, *P* < 0.001) between P-LOR2 and P-LOR3 (supplementary table 5, Supplementary Material online). These results support the hypothesis that LOR2 is distinct from other avian loricrins and was most likely lost in most lineages of birds following the divergence of Passeriformes from other crown birds (fig. 4).

## Conclusion

The results of this study demonstrate that loricrin genes are conserved within the EDC of birds and reptiles indicating that loricrin is an essential component of not only the mammalian CE (Hohl et al. 1991; Candi et al. 2005), but most likely of all amniotes. All loricrins identified were tandemly arrayed and found in the same orientation within the EDC between the genes EDQL (formerly EDQM3) and EDYM1. Although all species investigated had complete genome assemblies available on NCBI, the quality of the assemblies varied significantly (Jarvis et al. 2014; Zhang et al. 2014; supplementary table 2, Supplementary Material online). However, we were not able to find a relationship between the quality of loricrins and genome quality (results not shown). A frequent problem observed is the interruption of loricrin genes due to scaffold breaks (supplementary fig. 1, Supplementary Material online). Additionally, we encountered an abundance of unknown nucleotides interrupting the CDS of loricrin sequences resulting in artificial frameshifts. These problems are consistent with the results of previous studies (Milinkovitch et al. 2010; Hron et al. 2015; Peona et al. 2018) which have found that genome assemblers have difficulty resolving highly repetitive and GC rich regions of the genome, which can result in large numbers of gaps (i.e. fragmented sequences). Loricrins are highly repetitive which likely contributes to these problems. Similar problems have been encountered and resolved in other avian EDC genes through direct sequencing. Strasser et al. (2015) encountered a frameshift in the central domain of another avian EDC gene, the cysteine-rich EDCRP gene of the zebra finch. It has been demonstrated that EDCRP is expressed in the embryonic subperiderm of chickens as well as in the barbule cells of developing feathers (Strasser et al. 2015), which suggests it plays a role in the morphogenesis and structure of feathers and scales. Upon direct sequencing of zebra finch EDCRP, the frameshift was resolved and a single continuous open reading frame was identified (Strasser et al. 2015). Therefore, it is likely the frameshifts and premature stop codons observed in several loricrins are artificial and would be resolved upon direct sequencing.

The number of loricrin genes identified varied across different groups of organisms. Previous studies identified two loricrin genes in squamates, and only a single loricrin gene in turtles while the chicken has three loricrin (Holthaus et al. 2015, 2017; Strasser et al. 2014). Birds were the only group of species in our study which possessed three loricrin genes, whereas we were only able to identify a single copy of loricrin in crocodilian species. The results of our analysis of the crocodilian EDC are consistent with the recently published findings of Holthaus et al. (2018). We identified evidence of three loricrins in all bird species where the entire region of the EDC in which loricrins are located was assembled on a single scaffold with the exception of the pigeon, *C.livia*, where only two copies were identified (table 1). We did not identify any avian species that contained more than three copies or less than two copies of loricrin indicating that three copies of loricrin were most likely present in the most recent common ancestor (MRCA) of crown birds. These results, together with those of previous studies (Holthaus et al. 2015, 2017; Strasser et al. 2015), demonstrate a complex and dynamic duplication history of loricrins in birds and reptiles.

Our phylogenetic analyses identified four major clades of loricrins across birds and reptiles (fig. 3 and supplementary fig. 3, Supplementary Material online). In contrast to accepted comprehensive species phylogenies, crocodilian loricrins formed the outgroup to all other birds and reptiles, and the testudine loricrins grouped with avian LOR1 (Crawford et al. 2012; John 2012; Miller et al. 2012; Holthaus et al. 2018). These results demonstrate the evolutionary uncertainty described in previous studies (Crawford et al. 2012; John 2012; Holthaus et al. 2015) associated with defining the basal clade of all sauropsids. It is known that the epidermal appendages of birds and reptiles are highly specialized adaptations which exhibit significant molecular and genetic diversity even across phylogenetically similar species (Gremillet et al. 2005; Wang et al. 2016). It is possible that the results of our phylogenetic analyses reflect evolutionary adaptations associated with specialization of epidermal appendages such as crocodilian scales or the carapace of testudines, and are not indicative of the true phylogenetic history of birds and reptiles. These results suggest crocodilian loricrins have undergone little evolutionary divergence relative to those of birds and other reptiles. Additionally, these results suggest the possible convergent evolution of testudine loricrins with avian LOR1. Testudines, like birds, possess evolutionarily unique appendages in their shell and scutes, however unlike avian LOR1, testudines loricrins are ubiquitously expressed throughout the epidermis and its appendages (Strasser et al. 2014; Holthaus et al. 2015). The presence of NNNs in the loricrin sequences of both testudine species (Green sea turtle = 35.4% NNNs, painted turtle = 54.5% NNNs) may have impacted our phylogenetic results. Finally, PCA analysis demonstrated that the amino acid composition of avian LOR1 is distinct from that of testudine loricrins (fig. 6).

The LOR2/2B group of the second clade of avian loricrins contained only passerine loricrin sequences and LOR3 of the Hoatzin. Conversely, LOR2 of the Hoatzin grouped with other loricrins in the LOR3/LOR3B group. The nomenclature for Hoatzin loricrins, as all other species, was based on the genomic orientation of loricrins relative to other EDC genes (fig. 1). These data suggest a genomic inversion of LOR2 and LOR3 of the Hoatzin (fig. 1). We also found another, larger inversion in a different region of the turkey’s EDC indicating that inversions may be a major contributor to the evolution of EDC in (fig. 1A).

Our phylogenetic results support two likely scenarios for the evolution of avian loricrins (fig. 4). The first scenario entails the loss of an ancestral LOR2 from most orders of birds and its retention in Passeriformes, followed by recent lineage-specific duplications of LOR3 in most orders of birds. Alternatively, scenario 2 entails concerted evolution which has homogenized the LOR3/LOR3B and LOR2/LOR2B genes. Concerted evolution takes place when genes undergo gene conversion resulting in the homogenization of their DNA sequences (Sawyer 1989; Daiquing 1999). We found evidence of a statistically significant gene conversion event between LOR3 and LOR3B of the chimney swift (*C.pelagica*) (CHAPE BC KA, *P* = 0.00213) (supplementary table 3, Supplementary Material online). The likely concerted evolution in LOR3/LOR3B of the chimney swift, in combination with the absence of evidence supporting additional gene conversion events in other avian species suggest that a combination of concerted evolution, gene deletions, and gene duplications have shaped the evolution of avian loricrins.

In the first scenario of the evolution of avian loricrins (fig. 4), the recent gene duplications of LOR3 in most species analyzed resulted in the nearly identical LOR3/LOR3B and LOR2/LOR2B genes. Gene duplications have long been accepted as a major mechanism promoting evolutionary change (Holland et al. 1994). The most commonly observed mechanism of gene duplication, which occurs at high frequencies in birds, is unequal crossing over which generates tandem duplicates that are nearly identical in sequence and are genetically linked (Zhang 2003). The tandem linkage of avian loricrins is characteristic of gene duplications by unequal crossing over. Interestingly, previous studies (Dawson 2007; Backström 2010; Völker 2010) have provided evidence that recombination-based processes play a major role in avian evolution. This may correlate with the general absence of apparent loricrin “duplicates” (LOR3B/LOR2B) from all passerine birds except for the budgerigar (fig. 3). In the case of the budgerigar, LOR3 may have been lost, and instead LOR2 was duplicated into LOR2B. These results highlight the dynamic evolutionary nature of avian loricrins, even at the species level. Future studies which include additional loricrins will further elucidate if the similarities observed between avian LOR3/LOR3B and LOR2/LOR2B are primarily the result of recent gene duplications, concerted evolution, or the result of both mechanisms.

In mammals, loricrin functions as the major reinforcement protein of the CE, but also provides high levels of flexibility to the epidermis and its appendages (Hohl et al. 1991; Steinert et al. 1991). These key properties are thought to be achieved through a specialized conformation known as a Gly-Loop which results from the tandemly arranged, glycine-rich quasirepetitive peptide units which make up the central domain of loricrins. Existing solid-state NMR data have suggested that Gly-loop sequences are indeed highly flexible and that these sequences execute effectively isotropic motions (Karplus and Schulz 1985; Steven et al. 1989). These highly flexible loops consist of long stretches of primarily glycine and serine residues, but they do tolerate substitutions of other residues. These stretches of glycine and serine residues with occasional substitutions of polar residues are indexed upon aromatic and aliphatic residues which may associate to form a three-dimensional rosette-like array (Hohl et al. 1991; Steinert et al. 1991). Mammalian loricrins vary extensively in their size, exact organization and amino acid content, however they maintain the general form *x*(*y*)_*n*_ required for the formation of Gly-loops. This variation in mammalian Gly-loops is thought to play a major role in the mechanical properties conferred to the CE, such as flexibility, and tensile strength (Ishida-Yamamoto et al.1998). There are also known to be allelic variants of loricrin with slightly different amino acid compositions within individual populations which influence the properties of the epidermis and its appendages (Hohl et al. 1991; Steinert et al. 1991; Eckhart et al. 2013). Gly-loops provide their barrier function via weak hydrophobic interactions between the glycine and serine residues of adjacent Gly-loops, as well as other components of the CE such as keratins and filaggrin (fig. 5*A* and *B*). These interactions are thought to be easily interrupted upon application of stress which induces the formation of a separate but similar set of interactions. Once the stress is released, these new interactions are released to form yet another set of interactions similar but not identical to the original unstressed state. This is termed the “Velcro hypothesis” and accounts for the known flexibility and elastic recovery of the mammalian CE (Steinert et al. 1991).

Our results demonstrate that while there is significant variation across avian loricrins, they still adhere to the general form *x*(*y*)_*n*_ (fig. 2)_._ The observation that the sizes and sequences of avian Gly-loops are highly variable, but that the common structural motif of *x*(*y*)_*n*_ is conserved implies that the structural motif is more important for proper loricrin function than the exact sequence itself. A recent study using knockout mice without loricrin has demonstrated that without loricrin, the CE still assembles and functions, however several important components of the CE such as keratin-10 and -1 are incorporated at lesser amounts than in the wild-type and a mild phenotype is observed (Rice et al. 2016). These results indicate that loricrin plays an important role in CE assembly and structure by direct involvement with other proteins such as keratins 10 and 1, however it is not required for minimal CE barrier function. This suggests there may be little selective pressure on Gly-loop sequences which would allow them to tolerate considerable amounts of variation across different organisms. It is possible that this variation also contributes to the large amount of diversity observed in the feathers and scales of different species of birds; however more data is needed to identify any correlations between Gly-loop sequences and specific epidermal properties.

Previous studies have shown that variation in the number and proportion of β-keratin genes in birds correlates with different lifestyles such as predatory or aquatic (Greenwold et al. 2014). Similarly, the finding that the loricrins of different bird species contain highly variable numbers and sizes of Gly-loops could be correlated with the lifestyle or specific behaviors of avian species. Although our analysis was limited to a small number of species with high-quality loricrin sequences (table 2), we found that LOR3/3B of the chicken contain 43 and 48 Gly-loop domains, respectively, which is significantly higher than the 10 and 8 of LOR3/3B of the bald eagle. This may correlate with the predatory lifestyle of the bald eagle, whose beak and claws are more rigid and mechanically resilient structure for capturing and consuming prey than those of the chicken. Future studies using higher-quality loricrin sequences, potentially from direct sequencing, which examine the parameters of avian Gly-loops and look for correlation with avian lifestyles are needed to further evaluate this potential correlation.

The Gly-Loop domains of avian loricrins differ from those of mammals primarily in the identity of the aromatic/aliphatic amino acids upon which the loops are indexed. In mammals, these residues are primarily tyrosines, but there are occasional isoleucines, alanines, phenylalanines, and methionines. For example, the Gly-loops of mouse loricrin are indexed almost exclusively on tyrosine residues, whereas in human loricrin the loops are indexed on a combination of phenylalanine, tyrosine, isoleucine and valine residues. The general consensus repetitive unit of LOR1 is HQ(G/S)QGPICI(**G_x_**)SG which maintains the general form of *x*(*y*)_*n*_. The *I*soleucine (I) residues serve as long-chain aliphatic residues which are known to associate with one another to form a hydrophobic core, while the variable stretches of glycine and serine residues form the “loops” of the Gly-loop (fig. 4*A*). The sequence HQ(G/S)Q is conserved preceding the glycine-rich loop sequences. These glutamine residues are possibly involved in transglutamination via transglutaminases. In avian LOR1, the primary residue upon which loops are indexed are aliphatic isoleucines while in LOR2/3 the identity of these residues is more variable but primarily are tyrosines, isoleucines, and methionines. Furthermore, in avian loricrins, long-chain aliphatic residues are often found as dimers or trimers, whereas Gly-loops associated with aromatic amino acids are generally indexed upon only a single residue. This may result from the strength of the respective interactions. It is known that an extended row of aromatic residues is likely to stack in an ordered manner so that the phenyl rings align at a preferential distance and these interactions contribute 1–2.5 kcal/mol per aromatic pair toward the overall stability of the protein (Burley and Petsko 1985; Singh and Thornton 1985). In contrast, aliphatic residues do not by themselves associate to form highly ordered arrays, but it is well-known that they do associate to form a hydrophobic core. It is possible that the presence of multiple adjacent aliphatic residues aids in the association of aliphatic residues packing together to form a hydrophobic core (Rose and Roy 1980; Zhu et al. 1993).

Mammals possess a single loricrin gene which is preferentially crosslinked by different TGases throughout the process of cornification, whereas we found there are generally three loricrin genes in birds. It has been demonstrated that variation in the composition of amino acid residues which make up structural proteins, often correlates with different functionality (Candi et al. 2005). We analyzed the variation in amino acid content of the different avian loricrin genes and found that the amino acid contents of each respective amino acid in LOR1 were significantly different from those of other avian loricrins (supplementary tables 4 and 5, Supplementary Material online). Along with expression data from Strasser et al. (2014) which demonstrates LOR1 is differentially expressed in the chicken relative to LOR3/3B, these results indicate that the Gly-loops formed by the central domain of avian LOR1 likely have a unique functional role which is distinct from those of other loricrins. There were also significant differences in the amino acid compositions of LOR2 versus LOR3/LOR3B, specifically in cysteine, glycine, serine, and tyrosine contents all of which are known to be involved in the process of keratinocyte cornification (Eckhart et al. 2013; Rice et al. 2013). While we did observe that LOR3/LOR3B exhibited increased variation relative to LOR2, we contribute this to the fact that LOR2 is only found in Passeriformes while LOR3/LOR3B are represented by a much more diverse group of avian orders. There was no significant variation observed in the amino acid contents of type-B loricrins from their respective duplicates. This may be expected given that in the chicken, LOR3 and LOR3B have identical expression profiles in epidermal tissues (Strasser et al. 2014). Due to significant differences between the amino acid contents of LOR2 and LOR3/LOR3B, we predict that in passerine species LOR2 most likely exhibits a different expression profile than that of LOR3, and possibly a distinct function.

The feathers and scales of different bird species are novel adaptations which possess highly specialized properties which correspond to the diverse environments and lifestyles associated with birds. For example, the feathers of the great cormorant (*Nipponia nippon*) exhibit a unique morphological–functional adaptation to diving which balances the constraints of buoyancy and thermoregulation (Gremillet et al. 2005). The feathers of the Humboldt penguin (*Spheniscus humboldti*) exhibit unique hydrophobicity and anti-adhesion characteristics which endow them with excellent anti-icing properties and allow them to survive in arctic environments (Wang et al. 2016). Along with the variation previously described between different loricrin orthologs, we also observed interspecific variation in the amino acid contents of respective loricrin genes. This variation was most prevalent in LOR3, which was found in all species examined and is ubiquitously expressed in epidermal tissues (Strasser et al. 2014). This interspecific variation resembles that observed across mammalian loricrins, which is known to influence the mechanical properties endowed to the resulting CE (Hohl et al. 1991; Steinert et al. 1991). We propose that this variation in amino acid composition may correspond to specific evolutionary adaptations of feathers and other avian epidermal appendages. The least amount of interspecific variation in amino acid content was observed with LOR1 which interestingly is not expressed in feathers.

In mammals, loricrins are crosslinked primarily by the process of transglutamination via TGases. TGases catalyze the formation of N-(γ-glutamyl)-lysine isodipeptide bonds through the preferential, step-wise covalent cross-linking of glutamine and lysine residues located in both the N- and C- termini as well as interspersed throughout the central domain (Eckhart et al. 2013). We found that avian loricrins also possess several glutamine and lysine residues located at conserved positions. In LOR1, there are conserved glutamine residues in the H**Q**(G/S)**Q** portion of each repeat (fig. 5*A*). In LOR2 and LOR3, like mammalian loricrins, there are conserved glutamine residues in both the N- and C-termini that are located adjacent to lysines in the sequence QQK. There are also conserved lysine residues located near the aromatic/aliphatic residues upon which the glycine loops or indexed, furthermore these lysines are occasionally located adjacent to glutamine residues (fig. 5*B*).

Along with transglutamination, it is also known that disulfide bonding between adjacent cysteine residues plays a major role in facilitating the development of the epidermis and epidermal appendages in both mammals and birds (Hynes and Destree 1977; Kalinin et al. 2002). Strasser et al. (2015) characterized a cysteine-rich SEDC protein (EDCRP) in the chicken which is expressed in the subperiderm of feathers and scales. EDCRP consists of over 50% cysteine and most likely participates in disulfide bonding throughout epidermal development. Moreover, the cysteine content of several SEDC genes identified in the chicken exceeded 20% (Strasser et al. 2014). We identified adjacent cysteine residues located at conserved sites near the apex of many of the larger loricrin loops in avian LOR2 and LOR3. These residues potentially participate in disulfide bonding with other SEDC proteins such as EDCRP, other loricrins as well as various β-keratins. Disulfide bonding may also help facilitate the anchoring of loricrin and its associated proteins to the CE via interactions with SEDC genes similar to mammalian involucrin (Vanhoutteghem et al. 2008; Strasser et al. 2014). The presence of conserved adjacent cysteine residues throughout LOR2 and LOR3 suggest loricrins participate in not only transglutamination, but may also take part in a combination of covalent cross-linking interactions that result in the unique mechanical properties observed in feathers and other avian epidermal appendages.

Overall, the results of this study demonstrate a complex and dynamic evolutionary history of loricrins in archosaurs which likely involved gene duplications and deletions as well as concerted evolution and chromosomal inversions. The availability of more complete avian genomes is necessary to gain further insight into the evolution of avian loricrins. Given the conservation of the Gly-loop structure and expression profile of the loricrins in the chicken (Strasser et al. 2014), it is likely that avian loricrins constitute a major portion of the CE. Future studies which focus on a detailed expression profile of loricrins in other birds such as the passerines may provide further insight into the evolution of avian loricrin genes as well as the role they play in conferring the unique mechanical properties observed across the feathers of birds.

## Supplementary Material

Supplementary data are available at *Genome Biology and Evolution* online.

## Supplementary Material

Supplementary Data

## Notes

**Data deposition:** All genomic and sequence data information has been compiled and saved for any future reference.

## References

[evz054-B1] Alibardi L, et al 2016 Immunolocalization of a histidine-rich epidermal differentiation protein in the chicken supports the hypothesis of an evolutionary developmental link between the embryonic subperiderm and feather barbs and barbules. PLoS One 11(12):e0167789. 27936131 10.1371/journal.pone.0167789 PMC5147990

[evz054-B2] Alibardi L. 2017 Review: cornification, morphogenesis and evolution of feathers. Protoplasma 254(3):1259–1281. 27614891 10.1007/s00709-016-1019-2

[evz054-B3] Altschul SF, Gish W, Miller W, Myers EW, Lipman DJ. 1990 Basic local alignment search tool. J Mol Biol. 215(3):403–410. 2231712 10.1016/S0022-2836(05)80360-2

[evz054-B4] Altschul SF, et al 1997 Gapped BLAST and PSI-BLAST: a new generation of protein database search programs. Nucleic Acids Res. 25(17):3389–3402. 9254694 10.1093/nar/25.17.3389 PMC146917

[evz054-B5] Backström N. 2010 The recombination landscape of the zebra finch *Taeniopygia guttata* genome. Genome Res. 20:485–495. 20357052 10.1101/gr.101410.109 PMC2847751

[evz054-B6] Barati D, et al 2017 Synthesis and characterization of phot-cross-linkable keratin hydrogels for stem cell encapsulation. Biomacromolecules 18(2):398–412. 28000441 10.1021/acs.biomac.6b01493

[evz054-B7] Burley SK, Petsko GA. 1985 Aromatic-aromatic interaction: a mechanism of protein structure stabilization. Science 229(4708):23–28. 3892686 10.1126/science.3892686

[evz054-B8] Cai Q, Qian X, Lang Y. 2013 Genome sequence of ground tit *Pseudopodoces humilis* and its adaptation to high altitude. Genome Biol. 14(3):R29. 23537097 10.1186/gb-2013-14-3-r29 PMC4053790

[evz054-B9] Camacho C, et al 2009 Basic local alignment search tool. BMC Bioinformatics 10:42. 19821978 10.1186/1471-2105-10-329 PMC2770072

[evz054-B10] Candi E, Schmidt R, Melino G. 2005 The cornified envelope: a model of cell death in the skin. Nat Rev Mol Cell Biol. 6(4):328–340. 15803139 10.1038/nrm1619

[evz054-B11] Chuong CM. 2002 What is the ‘true’ function of skin? Exp Dermatol. 11(2):159–187. 11994143 10.1034/j.1600-0625.2002.00112.x PMC7010069

[evz054-B12] Crawford NG, et al 2012 More than 1000 ultraconserved elements provide evidence that turtles are the sister group of archosaurs. Biol Lett. 8(5):783–786. 22593086 10.1098/rsbl.2012.0331 PMC3440978

[evz054-B13] Daiquing L. 1999 Concerted evolution: molecular mechanism and biological implications. Am J Hum Genet. 64(1):24–30. 9915939 10.1086/302221 PMC1377698

[evz054-B14] Dawson DA. 2007 Gene order and recombination rate in homologous chromosome regions of the chicken and a passerine bird. Mol Biol Evol. 24(7):1537–1552. 17434902 10.1093/molbev/msm071

[evz054-B15] Eckhart L, Lippens S, Tschaler E, Declercq W. 2013 Cell death by cornification. BBA Mol Cell Res. 1833(12):3471–3480. 10.1016/j.bbamcr.2013.06.010 23792051

[evz054-B16] Ericson PGP, et al 2002 A Gondwanan origin of passerine birds supported by DNA sequences of the endemic New Zealand wrens. Proc Roy Soc B Biol Sci. 269(1488):234–241. 10.1098/rspb.2001.1877 PMC1690883 11839192

[evz054-B17] Fankl C, et al 2014 NCBI *Serinus canaria* annotation release 100. MPI Molgen, NCBI Eukaryotic Genome Annotation Pipeline. NCBI Genome GCF_000534875.

[evz054-B18] Gasteiger E, et al 2003 ExPASy: the proteomics server for in-depth protein knowledge and analysis. Nucleic Acids Res. 31(13):3784–3788. 12824418 10.1093/nar/gkg563 PMC168970

[evz054-B19] Gelman A, Rubin DB. 1992 Inference from iterative simulation using multiple sequences. Statist Sci. 7(4):457–511.

[evz054-B20] Greenwold MJ, et al 2014 Dynamic evolution of the alpha (α) and beta (β) keratins has accompanied integument diversification and the adaptation of birds into novel lifestyles. BMC Evol Biol. 14:249. 25496280 10.1186/s12862-014-0249-1 PMC4264316

[evz054-B22] Gremillet D, Chauvin C, Wilson RP, Meho YL, Wanless S. 2005 Unusual feather structure allows partial plumage wettability in diving great cormorants *Phalacrocorax carbo*. J Avian Biol. 36(1):57–63.

[evz054-B23] Hall TA. 1999 BioEdit: a user-friendly biological sequence alignment editor and analysis program for Windows 95/98/NT. Nucl Acids Symp. 41:95–98.

[evz054-B24] Hohl D, et al 1991 Characterization of human loricrin. J Biol Chem. 266(10):6626–6636. 2007607

[evz054-B25] Holland PWH, Garcia-Fernàndez J, Williams NA, Sidow A. 1994 Gene duplications and the origins of vertebrate development. Development 1994:125–133. 7579513

[evz054-B26] Holthaus KB, et al 2017 Identification and comparative analysis of the epidermal differentiation complex in snakes. Sci Rep. 7:45338. 28345630 10.1038/srep45338 PMC5366951

[evz054-B27] Holthaus KB, et al 2015 Comparative genomics identifies epidermal proteins associated with the evolution of the turtle shell. Mol Biol Evol. 33(3):726–737. 26601937 10.1093/molbev/msv265 PMC4760078

[evz054-B28] Holthaus KB, et al 2018 Comparative analysis of epidermal differentiation genes of crocodilians suggests new models for the evolutionary origin of avian feather proteins. Gen Biol Evol. 10(2):694–704. 10.1093/gbe/evy035 PMC5827346 29447391

[evz054-B29] Hron T, Pajer P, Pačes J, Bartůněk P, Elleder D. 2015 Hidden genes in birds. Genome Biol. 16:164. 26283656 10.1186/s13059-015-0724-z PMC4539667

[evz054-B30] Huelsenbeck JP, Ronquist F. 2001 MRBAYES: Bayesian inference of phylogeny. Bioinformatics 17(8):754–755. 11524383 10.1093/bioinformatics/17.8.754

[evz054-B31] Hynes RO, Destree A. 1977 Extensive disulfide bonding at the mammalian cell surface. Proc Natl Acad Sci USA. 74(7):2855–2859. 268636 10.1073/pnas.74.7.2855 PMC431319

[evz054-B32] Ishida-Yamamoto A, Takahashi H, Iizuka H. 1998 Loricrin and human skin diseases: molecular bases of loricrin keratodermas. Histol Histopathol. 13(3):819–826. 9690138 10.14670/HH-13.819

[evz054-B33] Jarvis ED, et al 2014 Whole genome analyses resolve the early branches in the tree of life of modern birds. Science 346(6215):1320–1331. 25504713 10.1126/science.1253451 PMC4405904

[evz054-B34] Jeanmougin F, Thompson JD, Gouy M, Higgins DG, Gibson TJ. 1998 Multiple sequence alignment with Clustal X. Trends Biochem Sci. 23(10):403–405. 9810230 10.1016/s0968-0004(98)01285-7

[evz054-B35] John JA. 2012 Sequencing three crocodilian genomes to illuminate the evolution of archosaurs and amniotes. Genome Biol. 13(1):415. 22293439 10.1186/gb-2012-13-1-415 PMC3334581

[evz054-B36] Karplus PA, Schulz GE. 1985 Prediction of chain flexibility in proteins. Naturwissenschaften 72(4):212–213.

[evz054-B37] Kalinin AE, Kajava AV, Steinert PM. 2002 Epithelial barrier function: assembly and structural features of the cornified cell envelope. BioEssays 24(9):789–800. 12210515 10.1002/bies.10144

[evz054-B38] Kumar S, Stecher G, Tamura K. 2016 MEGA7: molecular evolutionary genetics analysis version 7.0 for bigger datasets. Mol Biol Evol. 33(7):1870–1874. 27004904 10.1093/molbev/msw054 PMC8210823

[evz054-B39] Kypriotou M, Huber M, Hohl D. 2012 The human epidermal differentiation complex: cornified envelope precursors, S100 proteins and the ‘fused genes’ family. Exp Dermatol. 21(9):643–649. 22507538 10.1111/j.1600-0625.2012.01472.x

[evz054-B40] Milinkovitch MC, Helaers R, Depiereux E, Tzika AC, Gabaldón T. 2010 2x genomes – depth does matter. Genome Biol 11(2):R16. 20144222 10.1186/gb-2010-11-2-r16 PMC2872876

[evz054-B41] Miller HC, Biggs PJ, Voelckel C, Nelson NJ. 2012 De novo sequence assembly and characterization of a partial transcriptome for an evolutionarily distinct reptile, the tuatara (*Sphenodon punctatus*). BMC Genomics 13(1):439. 22938396 10.1186/1471-2164-13-439 PMC3478169

[evz054-B42] Notredame C, Higgins DG, Heringa J. 2000 T-Coffee: a novel method for fast and accurate multiple sequence alignment. J Mol Biol. 302(1):205–217. 10964570 10.1006/jmbi.2000.4042

[evz054-B43] Peona V, Weissensteiner MH, Suh A. 2018 How complete are ‘complete’ genome assemblies? – An avian perspective. Mol Ecol Resourc. 18:1188–1195. 10.1111/1755-0998.12933 30035372

[evz054-B44] Pierard G, Goffin V, Hermanns-Le T, Pierard-Franchimont C. 2000 Corneocyte desquamation. Int J Mol Med. 6(2):217–238. 10891569 10.3892/ijmm.6.2.217

[evz054-B45] Prum OR, et al 2015 A comprehensive phylogeny of birds (Aves) using targeted next-generation DNA sequencing. Nature 534:S7–S8. 10.1038/nature15697 26444237

[evz054-B46] Rambaut A. 2012 FigTree phylogenetic viewing software. http://tree.bio.ed.ac.uk/software/figtree/, last accessed June 14, 2018.

[evz054-B68] Rice RH, et al 2016 Proteomic Analysis of Loricin Knockout Mouse Epidermis. J Prot. Res. 15(8):2560–2566. 10.1021/acs.jproteome.6b00108 27418529

[evz054-B47] Rice RH, Winters BR, Durbin-Johnson BP, Rocke DM. 2013 Chicken corneocyte cross-linked proteome. J Proteome Res. 12(2):772–776. 10.1021/pr301036k PMC3569041 23256538

[evz054-B48] Robinson NA, Lapic S, Welter JF, Eckert RL. 1997 S100A11, S100A10, annexin i, desmosomal proteins, small proline-rich proteins, plasminogen activator inhibitor-2, and involucrin are components of the cornified envelope of cultured human epidermal keratinocytes. J Biol Chem. 272(18):12035–12046. 9115270 10.1074/jbc.272.18.12035

[evz054-B49] Ronquist F, Huelsenbeck JP. 2003 MRBAYES 3: Bayesian phylogenetic inference under mixed models. Bioinformatics 19(12):1572–1574. 12912839 10.1093/bioinformatics/btg180

[evz054-B50] Rose GD, Roy S. 1980 Hydrophobic basis of packing in globular proteins. Proc Natl Acad Sci USA. 77(8):4643–4647. 6933513 10.1073/pnas.77.8.4643 PMC349901

[evz054-B51] RStudio Team. 2015 RStudio. Integrated development for R. Boston (MA): RStudio, Inc Available from: http://www.rstudio.com/.

[evz054-B52] Sawyer S. 1989 Statistical tests for detecting gene conversion. Mol Biol Evol. 6(5):526–538. 2677599 10.1093/oxfordjournals.molbev.a040567

[evz054-B53] Singh J, Thornton JM. 1985 The interaction between phenylalanine rings in proteins. FEBS Lett. 191(1):1–6.

[evz054-B54] Stacklies W, Redestig H, Scholz M, Walther D, Selbig J. 2007 pcaMethods – a bioconductor package providing PCA methods for incomplete data. Bioinformatics 23(9):1164–1167. 17344241 10.1093/bioinformatics/btm069

[evz054-B55] Stamatakis A. 2014 RAxML version 8: a tool for phylogenetic analysis and post-analysis of large phylogenies. Bioinformatics 30(9):1312–1313. 24451623 10.1093/bioinformatics/btu033 PMC3998144

[evz054-B56] Steinert P, et al 1991 Glycine loops in proteins: their occurrence in certain intermediate filament chains, loricrins and single-stranded RNA binding protiens. Int J Biol Macromol. 13(3):130–139. 1716976 10.1016/0141-8130(91)90037-u

[evz054-B57] Steven AC, Mack JW, Trus BL, Bisher ME, Steinert PM. 1989 Cytoskeletal and Extracellular Proteins: Structure, Interactions and Assembly. Springer Verlag, Berlin p. 15.

[evz054-B58] Strasser B, et al 2014 Evolutionary Origin and Diversification of epidermal barrier proteins in amniotes. Mol Biol Evol. 31(12):3194–3205. 25169930 10.1093/molbev/msu251 PMC4245816

[evz054-B59] Strasser B, Mlitz V, Hermann M, Tschachler E, Eckhart L. 2015 Convergent evolution of cysteine-rich-proteins in feathers and hair. BMC Evol Biol 15:82. 25947341 10.1186/s12862-015-0360-y PMC4423139

[evz054-B60] Thompson JD, Gibson TJ, Plewniak F, Jeanmougin F, Higgins DG. 1997 The CLUSTAL_X windows interface: flexible strategies for multiple sequence alignment aided by quality analysis tools. Nucleic Acids Res. 25(24):4876–4882. 9396791 10.1093/nar/25.24.4876 PMC147148

[evz054-B61] Vanhoutteghem A, Djian P, Green H. 2008 Ancient origin of the gene encoding involucrin, a precursor of the cross-linked envelope of epidermis and related epithelia. Proc Natl Acad Sci Biol. 105(40):15481–15486. 10.1073/pnas.0807643105 PMC2563112 18809918

[evz054-B62] Völker M. 2010 Copy number variation, chromosome rearrangement, and their association with recombination during avian evolution. Genome Res. 20:503–511. 20357050 10.1101/gr.103663.109 PMC2847753

[evz054-B63] Wang H, et al 2000 In vitro assembly and structure of trichocyte keratin intermediate filaments: a novel role for stabilization by disulfide bonding. J Cell Biol. 151(7):1459–1468. 11134075 10.1083/jcb.151.7.1459 PMC2150680

[evz054-B69] Wang S, et al 2016 Icephobicity of penguins Spheniscus Humboldti and an artificial replica of penguin feather with air-infused hierarchical rough structures. J. Phys. Chem. 120:15923–15929.

[evz054-B64] Yang F, Zhao G, Zhou L, Li B. 2016 Complete mitochondrial genome of White-rumped Munia *Lonchura striata swinhoei* (Passeriformes: Estrildidae). Mitochondrial DNA 27(4):3028–3029. 26190080 10.3109/19401736.2015.1063052

[evz054-B65] Zhang G, et al 2014 Comparative genomics reveals insights into avian genome evolution and adaptation. Science 346(6215):1311–1320. 25504712 10.1126/science.1251385 PMC4390078

[evz054-B66] Zhang J. 2003 Evolution by gene duplication: an update. Trends Ecol Evol. 18(6):292–298.

[evz054-B67] Zhu BY, Zhou NE, Kay CM, Hodges RS. 1993 Packing and hydrophobicity effects on protein folding and stability: effects of β-branched amino acids, valine and isoleucine, on the formation and stability of two-stranded α-helical coiled coils/leucine zippers. Protein Sci. 2(3):383–394. 8453376 10.1002/pro.5560020310 PMC2142373

