# Supplementary material for: Complex Gene Loss and Duplication Events Have Facilitated the Evolution of Multiple Loricrin Genes in Diverse Bird Species
Source: Genome Biol Evol. 2019 Mar 13;11(3):984–1001. doi: 10.1093/gbe/evz054 (PMC6447390; doi:10.1093/gbe/evz054)
Supplement: Supplementary Data [file evz054_supp.zip › Supplemental material legends.docx]

**Supplementary Figure Captions**

**Supp. Figure 1 A and B – Example of a scaffold break interrupting the loricrin sequence of *Nestor notabilis.* (A)** Many loricrin sequences identified were incomplete or partial due to scaffold breaks and Unknown Nucleotides resulting in XXX’s. Scaffold NW_009939946.1 contains 17780 bases. EDQM3 (EDQL) and EDGH are found complete on this scaffold while LOR3 is prematurely terminated due to the end of the scaffold. The first half of LOR3 is found on the adjacent scaffold NW_009910415. **(B)** Furthermore, in LOR3B, a complete gene, there are several Unknown nucleotides within the central region which result in XXX’s in the amino acid sequence. Both of these were common problems encountered in loricrins as well as several other SEDC genes.

**Supp. Figure 2 – Gene organization of avian loricrins.** All avian loricrins share the same general organization as mammalian loricrins. They consist of highly conserved N- and C- terminal regions (N and C in figure), separated by a highly repetitive central region composed of a variable number of Glycine-rich repeat units. This figure depicts avian LOR1 genes, but this general organization is conserved across all other avian loricrin genes identified. Alignment scores pictured in top right; red/pink shades indicate high alignment scores, yellow indicates average alignment, and green/blue indicate poor alignment. Alignment wasa generated using T-Coffee (Notredame et al. 2000).

**Supp. Figure 3 – Bayesian Phylogenetic tree.** Phylogenetic tree produced using Baysian analyses. Tree largely in agreeance with Maximum-likelihood tree (Figure 2). Non-avian loricrins formed 3 distinct clades consisting of Mammals, Squamates, and crocodilian loricrin sequences respectfully. Bayesian analysis was in contrast to ML analysis and other currently accepted phylogenies of birds and reptiles by placing crocodilians as the basal group to all birds and reptiles with strong support. Our ML analysis had the same topography, but did not have high support values for this branch. Avian loricrins were organized into 2 major clades. The First, LOR1 clade included all terminal avian loricrins bordered by the SEDC EDYM1 as well as testudines loricrins as a sister group. The second avian clade was LOR2/LOR3 clade which consisted of 2 major sister groups of LOR3 and LOR2 respectfully. Only passerine birds and the Hoatzin possessed LOR2 loricrins. All species possessed a LOR3 loricrin, and all species except for Passeriformes, the hoatzin and Anna’s Hummingbird possessed a LOR3B gene organized in a lineage specific manner.
